# Solitary Peutz–Jeghers polyp harboring a focus of high-grade dysplasia in the colon: a case report and literature review

**DOI:** 10.1007/s12328-024-02059-x

**Published:** 2024-11-13

**Authors:** Takato Maeda, Tadashi Yoshizawa, Takao Oyama, Satoru Nakagawa, Yasuhisa Murai, Ryuma Machida, Nao Ishidoya, Juichi Sakamoto, Hideki Iwamura, Hirotake Sakuraba

**Affiliations:** 1 Department of Gastroenterology, Tsugaru General Hospital, Aomori, 037-0074 Japan; 2 Department of Gastroenterology and Hematology, Hirosaki University Graduate School of Medicine, Aomori, 036-8562 Japan; 3 Department of Pathology and Bioscience, Hirosaki University Graduate School of Medicine, Aomori, 036-8562 Japan; 4 Department of Gastroenterology, Aomori Prefectural Central Hospital, Aomori, 030-8553 Japan

**Keywords:** Solitary, Peutz–Jeghers polyp, Colon, Dysplasia

## Abstract

A solitary Peutz–Jeghers (PJ) polyp is a rare hamartomatous lesion without an associated PJ syndrome. However, little is known regarding malignancy arising in solitary PJ polyps. Here, we report a case of a solitary colonic PJ polyp with focal dysplasia. A 45-year-old asymptomatic man underwent total colonoscopy following a positive fecal occult blood test. The patient had no history of mucocutaneous pigmentation or family history of PJ syndrome. A 20 mm erythematous pedunculated polyp was observed in the sigmoid colon. Magnified endoscopy revealed a tubular or branch-like pit pattern with localized areas of irregular pits. These findings were suggestive of colorectal adenoma with high-grade dysplasia, and endoscopic mucosal resection was performed. Histopathological examination revealed arborizing proliferation of hyperplastic epithelia with smooth muscle bundles. In addition, a small number of irregular crypts with high-grade dysplasia were observed in the hyperplastic epithelium. Based on these histological findings, we finally diagnosed the patient with a solitary colonic PJ polyp with high-grade dysplasia. The present case suggests that solitary colonic PJ polyps may harbor dysplastic changes and require pathological evaluation with en bloc resection of the polyps.

## Introduction

Peutz–Jeghers (PJ) polyps are a type of hamartomatous polyps found in the gastrointestinal tract of patients with PJ syndrome [1, 2]. PJ polyps occasionally occur in patients without PJ syndrome and are classified as solitary PJ polyps [3]. PJ polyps are histologically characterized by the arborization of smooth muscle bundles with a hyperplastic epithelium [4]. Malignant transformation of solitary PJ polyps in the colon is rare; however, there are a few reports of polyps with dysplasia or adenoma [5–7], although this is controversial. Here, we report a case of a solitary colonic PJ polyp with high-grade dysplasia.

## Case report

**Fig. 1:**
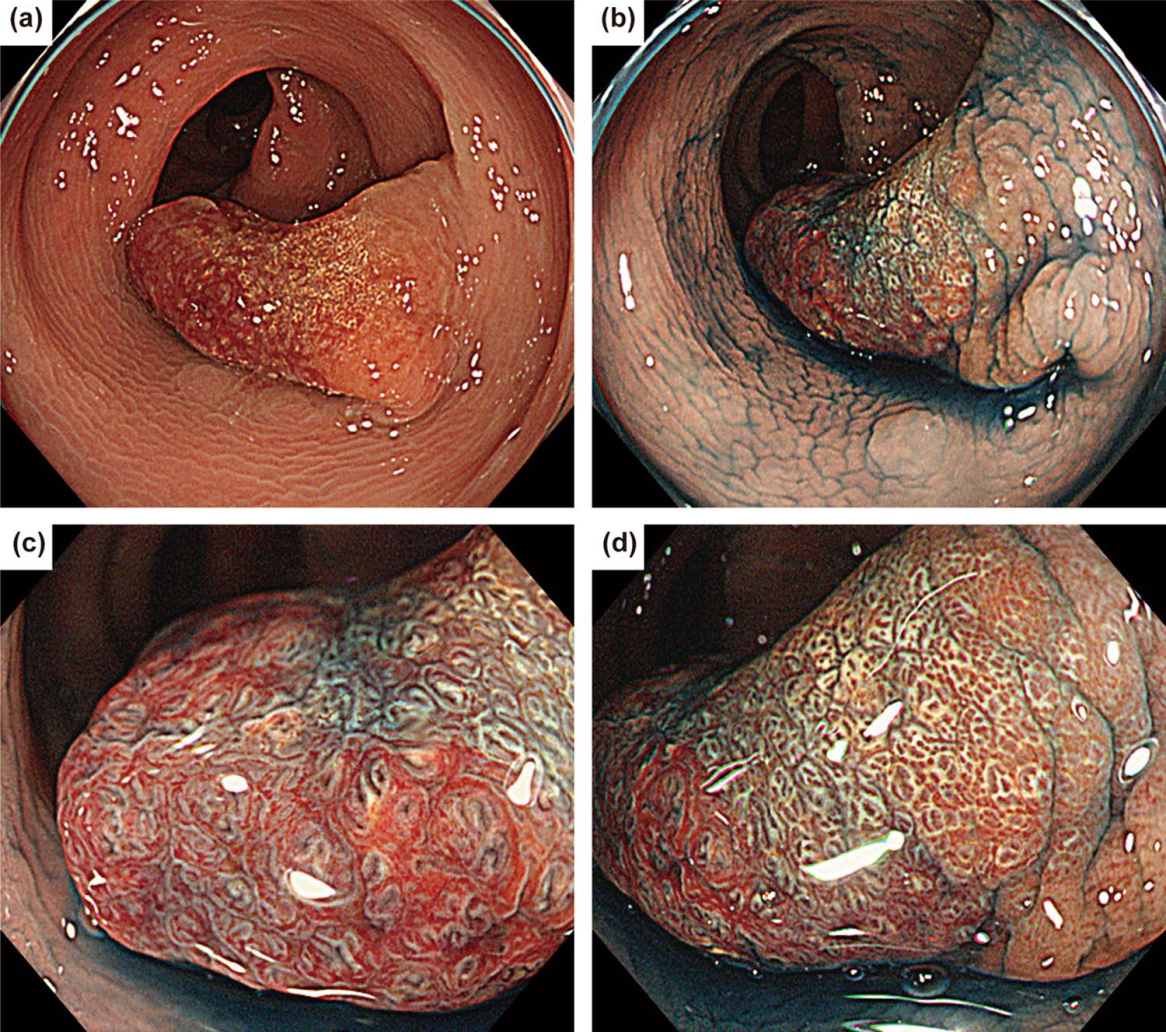
Endoscopic findings of the colonic polyp. **a** Colonoscopy shows a 20 mm pedunculated polyp in the sigmoid colon. **b** Indigo carmine spraying image. Magnified endoscopic images of the head (**c**) and base (**d**) of the polyp. An open or elongated pit is shown on the surface of the polyp

**Fig. 2:**
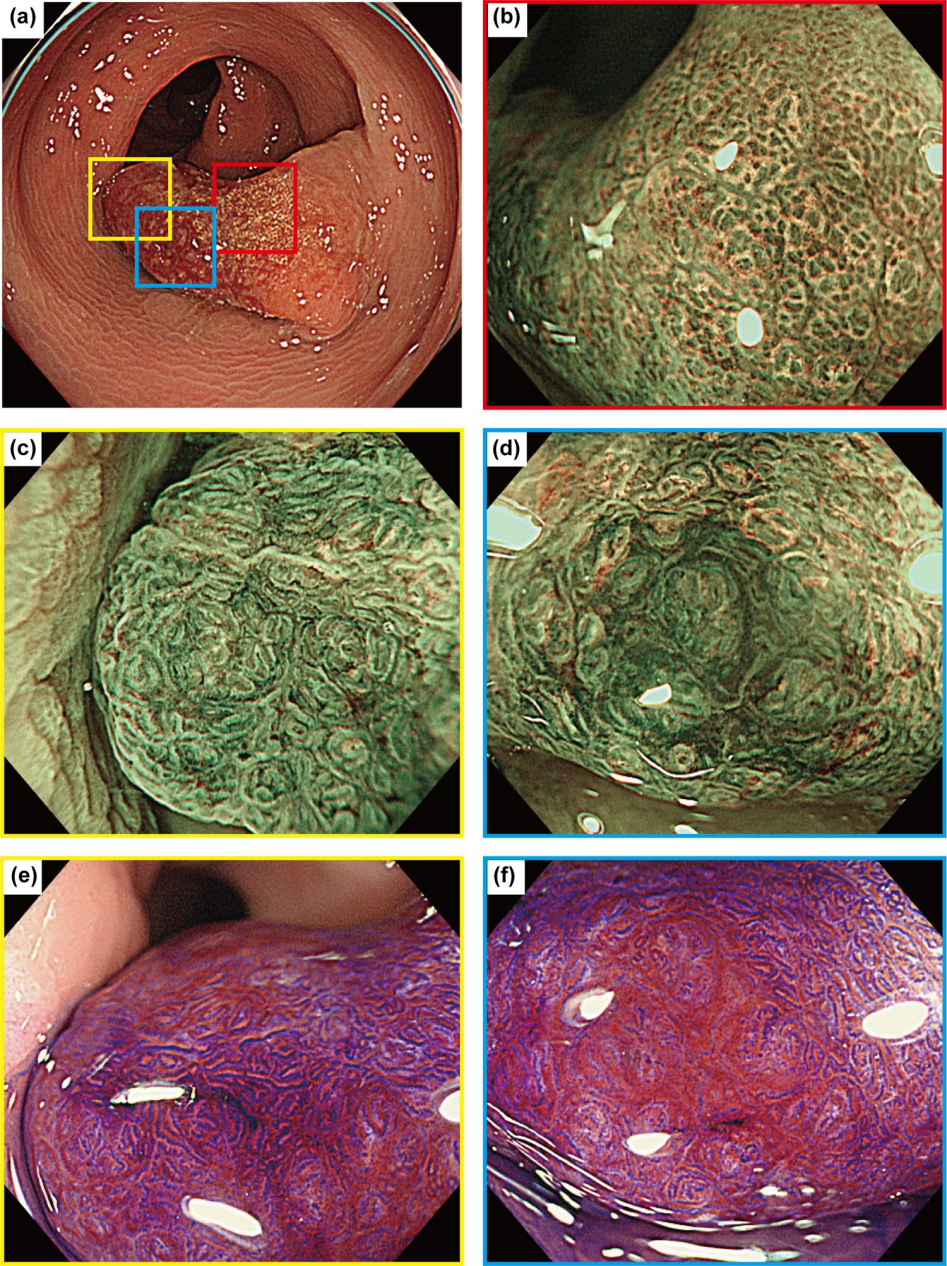
Magnified endoscopic images with narrow-band imaging and crystal violet staining. **a** Overall picture of the polyp. **b** The red box indicates the base of the polyp with a regular surface pattern. **c**, **e** The yellow box indicates the head of the polyp with a tubular or branched surface pattern. Cristal violet staining shows a mixture of type III_L_ and type IV pit patterns. **d**, **f** The blue box indicates the center of the polyp. The surface pattern is varied, with dense vessels in the intervening part. Cristal violet staining shows an area of indistinct pit structure

**Fig. 3:**
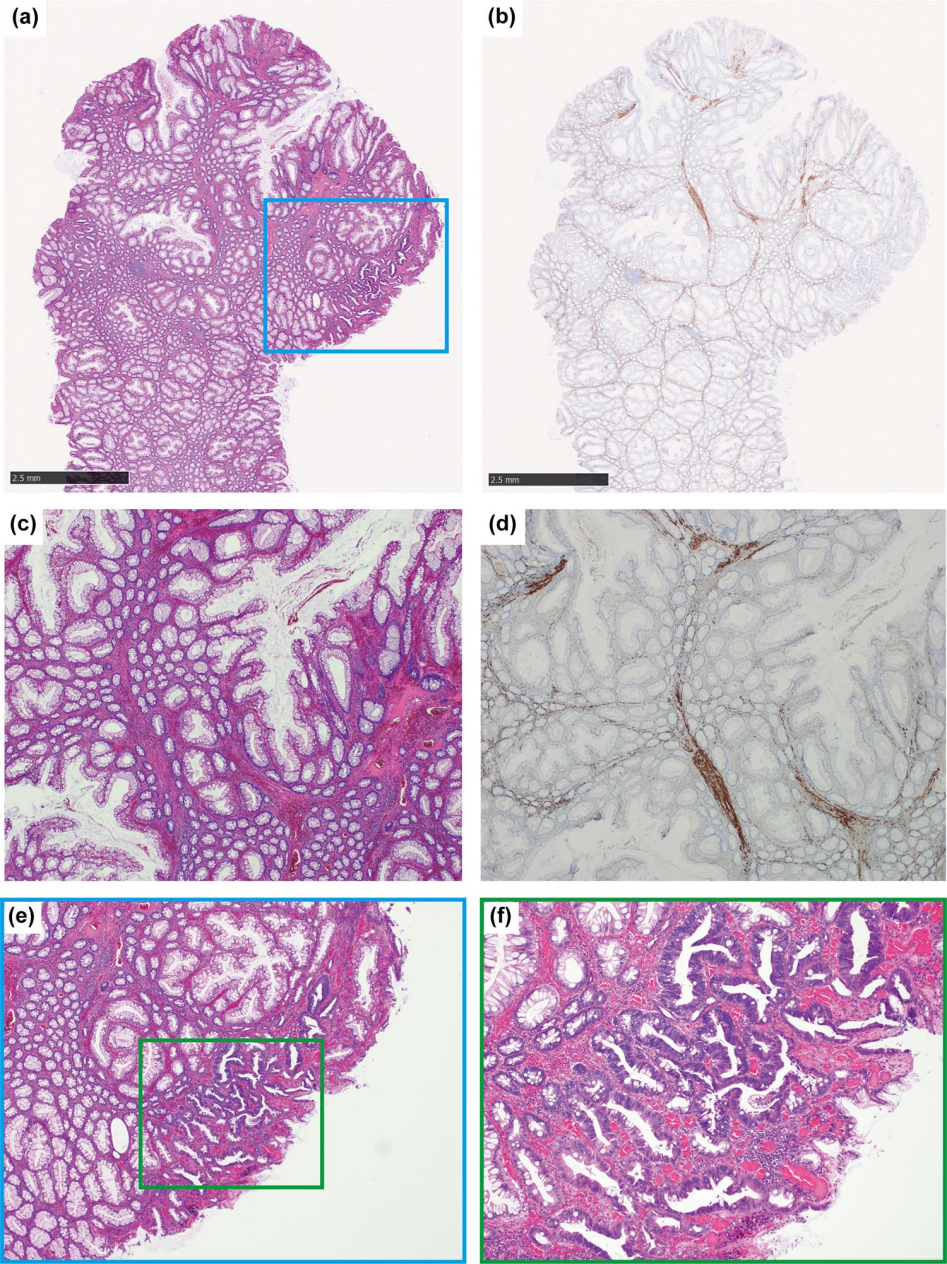
Microscopic findings of the excised polyp. **a**, **b** Histopathological loupe images of hematoxylin and eosin (HE) and desmin staining. **c**, **d** Low-power view of HE and desmin staining. Histopathological examination shows arborizing proliferation of smooth muscle bundles covered by hyperplastic epithelium. **e** Low-power view showing a focus of high-grade dysplasia within the epithelium (blue box in Fig. 3a). **f** High-power view showing high-grade dysplasia (green box in Fig. 3e)

A 45-year-old asymptomatic man was referred to our hospital for the evaluation of a positive fecal occult blood test and subsequent total colonoscopy. The patient had no history of mucocutaneous pigmentation or family history of PJ syndrome. A 20 mm erythematous raised lesion was observed in the sigmoid colon (Fig. 1). The head of the polyp was erythematous, and there was a yellowish-white area on the basal side and no clear demarcation between the polyp and surrounding mucosa. Indigo carmine spray revealed an open or elongated pit on the surface of the polyp (Fig. 1b–d). The intervening part of the pits was enlarged (Fig. 1c). Magnified endoscopy with narrow-band imaging revealed that the base of the polyp had a regular and cribriform-like surface pattern (Fig. 2b), and the head had a tubular or branched surface pattern (Fig. 2c). The center of the polyp had a varied surface pattern with dense vessels in the intervening part (Fig. 2d). Crystal violet staining showed that the polyp head had a mixture of type III_L_ and type IV pit patterns [8] (Fig. 2e). The center of the polyp had an area of indistinct pit structure (Fig. 2f). Based on these findings, we diagnosed the polyp as an adenoma-like lesion with high-grade dysplasia and performed an endoscopic mucosal resection. Histopathological examination revealed an arborizing proliferation of hyperplastic epithelia with smooth muscle bundles, consistent with a PJ-type hamartomatous polyp (Fig. 3a–d). The yellowish-white area on the basal side was consistent with a hyperplastic epithelium rich in goblet cells. Additionally, a small number of irregular crypts with high-grade dysplasia were observed within the hyperplastic epithelium (Fig. 3e, f). Although it was difficult to accurately contrast the endoscopic image with the pathological specimen, we inferred that the area of high-grade dysplasia was in the center of the polyp (Fig. 2d, f). No other polyps were found in the colon or rectum. Esophagogastroduodenoscopy and small bowel radiography performed on another day showed no abnormalities. Based on these findings, we diagnosed the patient with a solitary colonic PJ polyp, with a focus of high-grade dysplasia. We decided to perform a surveillance colonoscopy after one year.

## Discussion

In this report, we describe a rare case of solitary PJ polyp with dysplasia in the colon in which we were able to observe surface structure details. Focal high-grade dysplasia was found within the epithelium of the polyp, suggesting the need for pathological evaluation via en bloc resection of the solitary PJ polyp. To our knowledge, there are few endoscopic reports of a solitary colonic PJ polyp with high-grade dysplasia.

Solitary PJ polyps are less common in the colon. We conducted a literature search in PubMed for case reports published between 1996 and 2023. The following search terms were used: “solitary Peutz–Jeghers polyp” AND (“colon” OR “rectum”). We identified nine case reports [5–7, 9–14], which are summarized in Table 1. Among the nine cases, five had polyps in the sigmoid colon. The polyp was pedunculated in six cases. Moreover, in the study reported by Iwamuro et al., 39 of 51 solitary PJ polyps were found in the colorectum (21 in the sigmoid colon), and 40 cases showed a pedunculated morphology [15]. These findings characterize solitary PJ polyps as having a predilection for the sigmoid colon and a pedunculated morphology. In addition, the polyp heads were often reported to be lobulated (Table 1). A pedunculated polyp with a lobulated head may be characteristic of solitary PJ polyps; however, in this case, the head of the polyp was relatively smooth, and the stalk of the polyp was indistinct. These findings did not fit the previously reported characteristics of typical PJ polyps.

**Table 1:** A case study summary of solitary Peutz–Jeghers polyps in the colorectum

Year	Author	Age/sex	Reason for detection	Location	Morphology	Size	Endoscopic finding	Treatment	Dysplasia or adenoma
1996	Nakayama et al [9]	64/M	Lost weight	Rb	Pedunculated	20 mm	NL	Surgery	None
2005	Jaremko et al [10]	19/M	Intussusception	D	NR	NR	NL	Surgery	None
2009	Itaba et al [11]	50/M	Screening	S	Pedunculated	18 mm	Lobulated with grooves	Loop diathermy	None
2011	Limaiem et al [5]	27/F	Bleeding	Rb	Sessile	150 mm	Lobular polypoid lesion	Polypectomy	Adenoma
2013	Tsujii et al [6]	46/M	NR	S	Pedunculated	30 mm	Lobulated, SMT-like nodule on the stalk	EMR	Tubulovillous adenoma
2017	Papalampros et al [7]	48/F	Abdominal distention, pain, hemorrhage	S	NR	NR	NL	Surgery	High-grade dysplasia
2017	Linhart et al [12]	77/M	Midabdominal pain	S	Pedunculated	30 mm	NL	Polypectomy	None
2019	Putra et al [13]	17/M	NR	T	Pedunculated	20 mm	Irregular erythematous mucosa	NR	None
2021	Oluyemi et al [14]	58/M	Screening	S	Pedunculated	NR	Lobulated	Polypectomy	None

*NR* none reported, *NL* none listed, *Rb* lower rectum, *S* sigmoid colon, *D* descending colon, *T* transverse colon, *SMT* submucosal tumor, *EMR* endoscopic mucosal resection

Solitary PJ polyps are often difficult to distinguish from colorectal adenomas by endoscopy. Tsujii et al. reported large tubular or gyrus-like pit patterns on magnified endoscopic observations of solitary colonic PJ polyps [6]. In our case, the polyp had a tubular or branch-like pit pattern, and we diagnosed it as an adenoma-like lesion. However, the observed findings, such as the mixture of different surface patterns and enlargement between pits, differed from those of typical adenomas and may indicate a point of differentiation between adenomas and solitary PJ polyps. In addition, dilated glandular orifices were conspicuous when indigo carmine was sprayed, suggesting dilation of the glandular ducts due to epithelial hyperplasia of the mucosa, which is a pathological feature of PJ polyps. Further studies are needed to clarify the endoscopic features of solitary PJ polyps.

One clinical implication of this case arises because solitary PJ polyps can harbor dysplasia. Previous studies on the malignant potential of solitary PJ polyps have been controversial. Several case series have reported no recurrence after the resection of solitary PJ polyps and a low risk of cancer morbidity [15–17]. However, these studies also included solitary PJ polyps that developed in gastrointestinal tracts other than the colon. In contrast, in three of nine case reports of solitary PJ polyps in the colon, the polyps contained adenomas or high-grade dysplasia (Table 1). In a study investigating syndromic and sporadic PJ polyps, 7 of 87 (8.0%) sporadic PJ polyps had dysplasia, at a significantly higher rate than that observed in syndromic PJ polyps [18]. In addition, a methylome analysis showed potential features of the colorectal cancer epigenome in a solitary PJ polyp [12]. These findings suggest that solitary PJ polyps may harbor dysplastic changes and require pathological evaluation via en bloc resection. However, the incidence of invasive cancer, recurrence, and appropriate surveillance intervals for solitary PJ polyps with dysplasia have not been clearly reported and should be clarified in further case series.

In conclusion, solitary PJ polyps are rare colorectal polyps and may harbor dysplasia. Solitary colonic PJ polyps should be resected en bloc and pathologically evaluated for dysplasia.
